# Endogenous CRISPR‐Based Removal of Tetracycline Resistance in *Bifidobacterium animalis* subsp. *lactis* Through a Safe‐by‐Design Approach

**DOI:** 10.1111/1751-7915.70443

**Published:** 2026-09-15

**Authors:** Marianna Bozzetti, Matteo Raneri, Claudia Cortimiglia, Giovanni Milani, Federica Volontè, Franco Lucchini, Daniela Bassi, Pier Sandro Cocconcelli

**Affiliations:** ^1^ Department for Sustainable Food Process—DiSTAS Università Cattolica del Sacro Cuore Cremona Italy; ^2^ Sacco srl Cadorago Como Italy; ^3^ Institute of Advanced Science for Agriculture Jolanda di Savoia (FE) Italy

**Keywords:** antimicrobial resistance gene (ARG), *Bifidobacterium animalis* subsp. *lactis*, CRISPR‐Cas, genome editing, probiotic, Safe‐by‐Design approach, tetracycline

## Abstract

*Bifidobacterium animalis* subsp. *lactis* is widely used as a probiotic; however, the presence of the tetracycline resistance gene *tetW* raises safety and regulatory concerns due to its potential mobility within the gut microbiome. Here, we applied a Safe‐by‐Design strategy using the endogenous CRISPR‐Cas system of *B. animalis* subsp. *lactis* BLC01 to inactivate *tetW* through the introduction of premature stop codons. Whole‐genome sequencing confirmed the intended editing and excluded relevant off‐target effects. *tetW* inactivation markedly reduced the tetracycline minimum inhibitory concentration, restoring susceptibility below the tetracycline cut‐off value for bifidobacteria (8 μg/mL). Comparative phenotypic analyses demonstrated that the edited strain (BLC01‐2F3G10) retained key probiotic traits, including tolerance to acid, bile, and osmotic stress, exopolysaccharide production, aggregation capacity, survival during simulated gastrointestinal digestion and adhesion to intestinal epithelial cells. Importantly, no reversion to tetracycline resistance was observed after prolonged exposure to sub‐inhibitory minimal selective antimicrobial concentration, indicating genetic stability of the edited phenotype. Collectively, these findings demonstrate that endogenous CRISPR‐based genome editing can be leveraged to selectively remove antimicrobial resistance determinants from probiotic strains while preserving functionality, supporting the development of next‐generation probiotics with an improved safety profile and reduced potential for antimicrobial resistance dissemination in the human gut.

## Graphical abstract

Endogenous CRISPR–Cas editing was used to inactivate *tetW* gene in *Bifidobacterium animalis* subsp. *lactis*. The engineered strain showed reduced tetracycline resistance while retaining key probiotic traits and genetic stability. This work demonstrates a Safe‐by‐Design strategy for engineering safer industrial probiotics and mitigating antimicrobial resistance in beneficial bacteria.

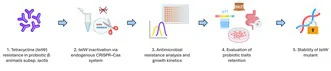

## 1 Introduction

Species of the genus *Bifidobacterium* are among the most extensively studied and widely applied probiotics, known for their crucial roles in modulating gut microbial communities, enhancing intestinal barrier integrity and regulating host immune responses (Bocchio et al. 2024). As some of the earliest colonizers of the human gastrointestinal tract, *Bifidobacterium* spp. predominates in both infants and healthy adults (Bocchio et al. 2024). Among the different members of the *Bifidobacterium* genus, *B. animalis* subsp. *lactis* is the most commonly used as probiotic in food and food supplements. This microorganism has demonstrated multiple health‐promoting effects, including improvement of colonic barrier function (Krumbeck et al. 2018), mitigation of the adverse effects of antimicrobial treatment (Merenstein et al. 2021), enhancing oral health (Araujo et al. 2022; Oliveira et al. 2017), alleviation of symptoms associated with low defecation frequency and infant colic (Collins et al. 2025) and cholesterol‐lowering activity (Yang et al. 2024). However, phylogenomic and in silico analyses reveal that the tetracycline resistance gene *tetW*, which encodes a ribosomal protection protein that prevents tetracycline binding to the ribosome, is widely distributed among strains of this subspecies (Gueimonde et al. 2010; Markley and Wencewicz 2018; Nøhr‐Meldgaard et al. 2021). Moreover, this gene is frequently flanked by mobile genetic elements, thereby posing a potential risk for horizontal gene transfer (Kazimierczak et al. 2006; Rozman et al. 2020; Wang et al. 2017). Comparative sequence analysis of *tetW* loci from 10 human intestinal *Bifidobacterium* strains revealed 98%–100% identity within a 2.1 kb core region, with several flanking segments containing insertion‐sequence elements indicative of potential mobility (Ammor et al. 2008). The same previously reported 2154 bp segment was further detected inside a conserved 2281 bp *tetW* sequence in 21 human‐derived *Bifidobacterium* strains across four species (Wang et al. 2017). Conserved sequences found in bifidobacteria genomes showed high nucleotide identity (98%–100%) with *tetW*, *tetO* and *tetS* loci found in various human commensal and pathogenic bacteria belonging to *Arcanobacterium*, *Streptococcus*, *Corynebacterium*, *Campylobacter* and *Listeria* genera (Wang et al. 2017). Moreover, the *tetW* genetic locus has a CG content of 51.9%, which is considerably lower than the overall GC content of the BLC01 genome (60.5%) (Bottacini et al. 2011; Rozman et al. 2020). Collectively, these findings are consistent with the horizontal acquisition of *tetW* through gene transfer events. According to the European Food Safety Authority (EFSA) guidelines, any ARG is considered a hazard and may preclude Qualified Presumption of Safety (QPS) status unless its intrinsic nature is demonstrated (Koutsoumanis et al. 2023). Recent metagenomic analyses of commercial probiotic products have detected more than 70 distinct ARGs, including hybrid *tet(W/N/W)*, frequently linked to integrative conjugative elements (Tóth et al. 2021). Given the potential public health concerns associated with mobile ARGs in probiotic strains, targeted strategies to eliminate or mitigate these elements are essential to ensure the safety and regulatory compliance of commercial probiotic products.

Genome editing (GE) has been proposed as a promising approach for the genetic improvement of bacterial strains (Börner et al. 2018; Mullins et al. 2024). The CRISPR‐Cas system is classified as New Genomic Techniques (NGTs) defined in the Regulation (EU) 2026/1388 as ‘a diverse group of techniques, that can result in organisms with modifications equivalent to those which can be obtained by conventional breeding methods or in organisms with more complex modifications’ (European Parliament and Council 2026). Although this initiative is currently restricted to the plant sector, it marks a fundamental regulatory trend that could lead to a similar, simplified regulatory framework for NGT microorganisms in the future. According to EFSA, the application of NGTs to microorganisms does not pose novel hazards to humans, animals or the environment when compared to Established Genomic Techniques (EGTs) or conventional mutagenesis, with respect to the technique itself (Mullins et al. 2024). The concept of *Safe‐by‐Design* (SbD) has been introduced to encourage the integration of safety considerations from the earliest stages of development (Kallergi and Asveld 2024). Recently, EFSA outlined strategies for assessing the safety of bacteria modified using NGTs (Bennekou et al. 2025). The key concepts are the comparative approach, based on the substantial equivalence between the parental non‐modified strain and the strain resulting from the genetic modification, the analysis of the effect of intended and unintended genetic modification (GM), and the impact of the newly introduced traits on the environment, by analysing the different areas of risks. This approach was designed to consent to a risk assessment of released bacteria derived from NGTs in different environments, including foods and the human and animal gut. Key risk areas include persistence and invasiveness, horizontal gene transfer and effects on target organisms.

The CRISPR‐Cas system has emerged as one of the most versatile and precise GE tools available for microbial engineering (Ding et al. 2020). Originally discovered as an adaptive immune mechanism in bacteria and archaea, CRISPR‐Cas has been redesigned to achieve targeted gene disruption, deletion and replacement across a wide range of organisms (Duan et al. 2021). In particular, the CRISPR–Cas9 system represents a promising strategy to eliminate ARGs in bacteria (de la Fuente Tagarro et al. 2024). Within the genus *Bifidobacterium*, endogenous and heterologous CRISPR/Cas systems have recently been employed to enable precise GE (Li et al. 2023; Lin et al. 2025). However, the widespread presence of various restriction–modification systems in bifidobacteria presents a major barrier to genetic manipulation, as these systems actively degrade exogenous DNA, thereby reducing transformation efficiency and compromising plasmid stability (Han et al. 2022). Despite these challenges, genome mining reveals that 54%–77% of *Bifidobacterium* genomes encode CRISPR/Cas arrays, primarily of the Type I subtype, which provide potential in situ GE platforms (Briner et al. 2015; Han et al. 2022). Notably, Han et al. (2024) employed the endogenous Type I‐C system in *B. breve* FJSWX38M7 to inactivate the *upp* gene via a target single‐base substitution and the insertion of three premature stop codons. In a similar approach, Pan et al. (2022) repurposed the endogenous Type I‐G CRISPR‐Cas system in *B. animalis* subsp. *lactis* to delete a large genomic region encompassing the *tetW* gene.

This study aims to evaluate the safety and the physiological traits of relevance for probiotic activity of a *B. animalis* subsp. *lactis* BLC01 in which the *tetW* gene has been edited via the endogenous CRISPR‐Cas system, thereby eliminating the tetracycline resistance trait. We firstly analysed the effects of editing on tetracycline phenotype and then we performed a risk assessment of the new probiotic derivative BLC01‐2F3G10, based on the comparative analysis between the parental and the CRISPR‐edited derivative strain. Collectively, the analysis of phenotypic traits included the tolerance to acid, bile, osmotic stress and simulated gastric and intestinal fluids, exopolysaccharides (EPS) production, auto‐ and co‐aggregation capacity, survival during in vitro gastrointestinal digestion and adhesion to intestinal epithelial cells. Furthermore, we aimed to use this case as a model to assess the impact of NGTs on the long‐term stability of an edited strain in the presence of Minimum Selective Concentration (MSC) of tetracycline. This integrative approach aims to validate that the edited strain retains its full probiotic potential while exhibiting an enhanced safety profile aligned with regulatory standards.

## 2 Materials and Methods

### 2.1 Bacterial Strains, Plasmids and Growth Conditions

The bacterial strains employed in this study included *Bifidobacterium animalis* subsp. *lactis* BLC01 (Sacco System, Cadorago, Italy) and *Escherichia coli* NEB10‐beta (New England Biolabs, Ipswich, MA, USA), with the latter serving as the cloning host. *B. animalis* subsp. *lactis* was grown in De Man Rogosa Sharpe (MRS) broth (Oxoid, Basingstoke, UK) supplemented with 0.05% (w/v) L‐Cysteine Hydrochloride (Merck KGaA, Darmstadt, Germany) at 37°C. *E. coli* was cultured in Luria‐Bertani (LB) broth (Merck KGaA, Darmstadt, Germany) at 37°C.

The *E. coli–Bifidobacterium* shuttle vector pAM1 was used for plasmid construction (Álvarez‐Martín et al. 2008). A comprehensive list of the plasmids used in this study is provided in Table 1.

**TABLE 1:** List of plasmids used in this study.

Plasmid	Description	References
pAM1	*Escherichia coli* –*Bifidobacterium* shuttle vector pAM1; Erm^R^	Álvarez‐Martín et al. (2008)
pAM1‐preT BLC01	Pre‐targeting plasmid with an artificial crRNA (leader from the native BLC01 CRISPR array + two CRISPR direct repeats + rho‐independent terminator BBa_B1006) cloned into pAM1	This study
pAM1‐tetW CRISPR	Targeting plasmid on the BLC01 *tetW* gene generated after cloning a 33‐nt spacer into pAM1‐ preT BLC01	This study
pAM1‐tetW rep	Editing plasmid obtained by cloning in pAM1‐tetW CRISPR the repair template used to insert three consecutive stop codons in the *tetW* gene	This study

Bacterial transformants were selected in Brain Heart Infusion (BHI) agar (Merck KGaA, Darmstadt, Germany) supplemented with 150 μg/mL erythromycin for *E. coli* , and on MRS agar containing 0.05% (w/v) L‐cysteine and 2.5 μg/mL erythromycin (Merck KGaA, Darmstadt, Germany) for *B. animalis* subsp. *lactis* under anaerobic conditions.

For co‐aggregation assays, *E. coli* ATCC 25922 and *Salmonella enterica* subsp. *enterica* UC3605 were used in combination with *B. animalis* subsp. *lactis* strains. These strains were cultured overnight in BHI broth (Oxoid, Basingstoke, UK) at 37°C.

As control strains in adhesion assays to human intestinal cell lines, *Lacticaseibacillus rhamnosus* ATCC 53103 (GG) and *Lactobacillus delbrueckii* subsp. *lactis* DSM 2072 were employed. Both control strains were cultured overnight in MRS broth at 37°C.

### 2.2 Molecular Techniques and Construction of BLC01 *tetW* Negative Mutants

The CRISPR locus of *B. animalis* subsp. *lactis* BLC01 was identified using CRISPRCasFinder (Couvin et al. 2018). Spacer sequences were extracted and analysed with CRISPRTarget (Biswas et al. 2013), by searching the IMG/VR database (Paez‐Espino et al. 2017) to determine protospacer‐adjacent motif (PAM) sequences. Consensus PAM sequences were generated using the WebLogo tool (Crooks et al. 2004). Genomic DNA was extracted using the UltraClean Microbial DNA Isolation Kit (Qiagen, Hilden, Germany), while plasmid DNA from *E. coli* strains was purified using the QIAprep Spin Miniprep Kit (Qiagen, Hilden, Germany), in accordance with the manufacturers' instructions. PCR primers and single‐stranded DNA (ssDNA) oligonucleotides were synthesized by Eurofins Genomics GmbH (Ebersberg, Germany), and synthetic DNA fragments for CRISPR RNA (crRNA) expression and homology‐directed repair were obtained from GenScript Biotech Corporation (Nanjing, China) and Twist Bioscience (South San Francisco, CA, USA), respectively. Phusion High‐Fidelity DNA Polymerase and DreamTaq DNA Polymerase (Thermo Fisher Scientific, Waltham, MA, USA) were used for cloning and construct verification, respectively. The list of PCR primers used in this study is provided in Table 2. PCR amplicons were analysed on 1% (w/v) agarose gel, and cloned fragments were subsequently verified by Sanger sequencing (Microsynth AG, Balgach, Switzerland). Restriction digestion was performed with 1 μg of DNA in a 20 μL reaction volume at 37°C for 1–3 h, using high‐fidelity enzymes (New England Biolabs, Ipswich, MA, USA). Digested products were purified with the QIAquick PCR & Gel Cleanup Kit (Qiagen, Hilden, Germany). Ligation was performed using T4 DNA Ligase (New England Biolabs, Ipswich, MA, USA), with 50 ng of vector DNA and an insert‐to‐vector molar ratio of 3:1 to 5:1, in a total volume of 20 μL. Annealing of ssDNA oligonucleotides (100 μM) was performed by mixing 2 μg of each strand in 50 μL of buffer (10 mM Tris, pH 7.5–8.0; 50 mM NaCl; 1 mM EDTA), followed by heating at 93°C for 3 min and gradual cooling to room temperature. Annealed oligonucleotides were stored at −20°C and used at a 1:10 dilution for downstream cloning applications.

**TABLE 2:** List of PCR primers used in this study.

Primer name	Sequence (5′–3′)
FW pAM1	GTTGTAAAACGACGGCCAGTG
RV tetW preT	AGCGCTTTTGCGAGAGGTAGA
sp1 tetW top^a^	AGGGCATGCTTTTGGAGCGGCAGCGTGGGATTACCATA
sp1 tetW botA^a^	GAGATATGGTAATCCCACGCTGCCGCTCCAAAAGCATG
RV sp1 tetW	ATGGTAATCCCACGCTGCCG
RV tetW rep	TGGACATTTGCCGGTGTAGC
FW tetW US	GATTCCAGCAACCAGGTTC
RV tetW DS	GTGGGTTCCTTTACCACTG
hsGlob_Fw	GCTGCTGGTGGTCTACCCTTG
hsGlob_rev	GCACTTTCTTGCCATGAGCCT
Uni16S_fw	GGTTAAGTCCCGCAACGA
Uni16S_rev	TCATCCCCACCTTCCTCC

^a^ Annealing oligonucleotides to generate the *tetW*‐targeting spacer.

**FIGURE 1:**
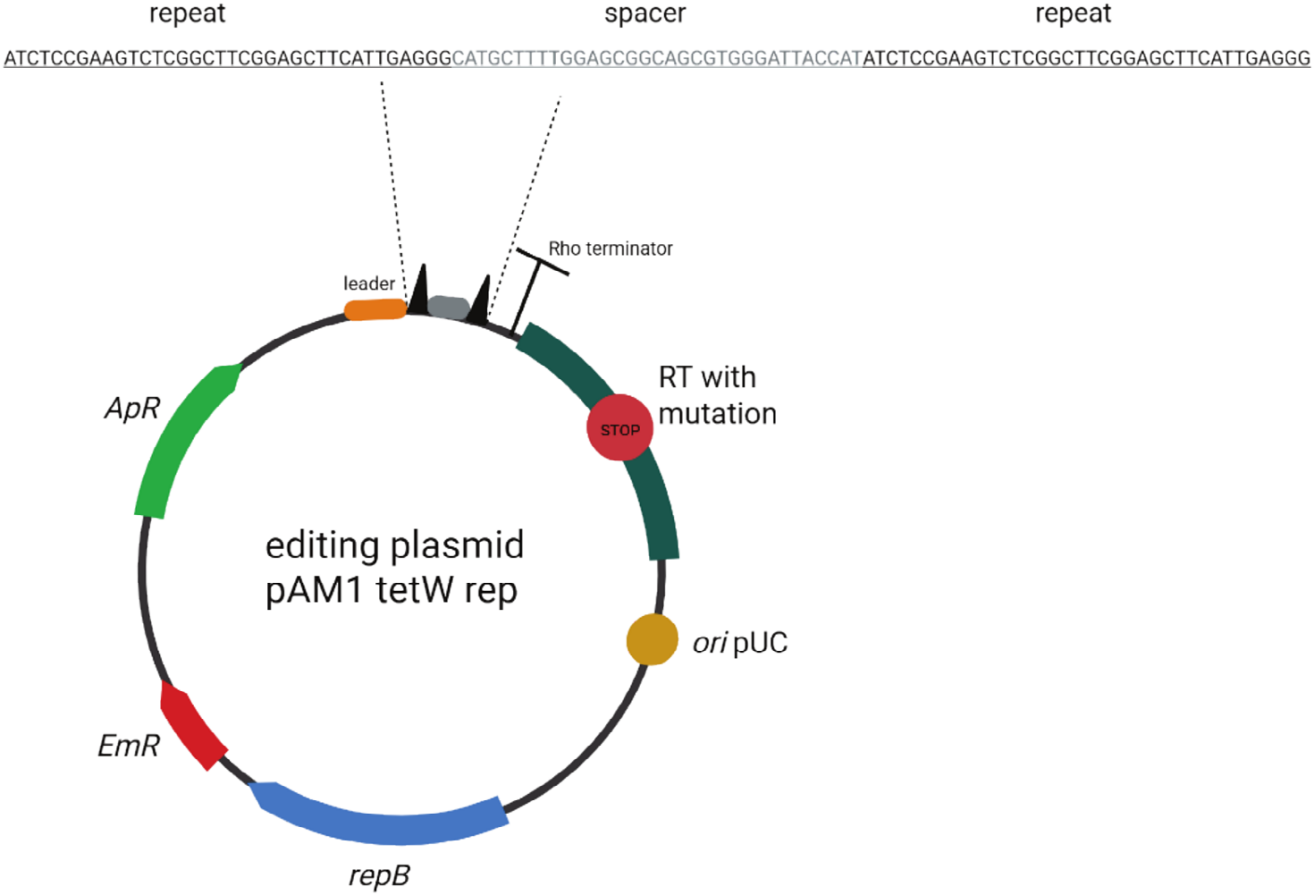
Schematic representation of the editing plasmid constructed to inactivate the *tetW* gene in BLC01. The DNA construct used to target the native CRISPR‐Cas system of BLC01 to *tetW* encompasses the leader region of BLC01 CRISPR (in orange), two CRISPR direct repeats (in black) flanking the self‐targeting *tetW* spacer (in grey) and a Rho‐independent transcription terminator (T structure). The subsequent cloning of 2 kb repair template (RT; in dark green), carrying the desired mutations on *tetW* (red dot), promotes BLC01 genome editing on *tetW* by homology‐directed DNA repair.

The editing plasmid, designed to reprogram the endogenous CRISPR‐Cas system of BLC01 to silence the *tetW* gene, was assembled by ligating pAM1 with two synthetic DNA fragments: a fragment coding for the crRNA targeting *tetW* and a 2 kb homology‐directed repair template (RT) containing 1 kb upstream and downstream homology arms flanking the selected *tetW* protospacer, together with mutations introducing three consecutive premature stop codons in *tetW* (Figure 1). The artificial crRNA was cloned into a *Bgl*II‐*Sal*I digested pAM1 vector, generating pAM1‐preT BLC01. It contains a promoter region from the native BLC01 CRISPR array, two CRISPR direct repeats, and a rho‐independent terminator (BBa_B1006). The ligation mix was transformed into chemically competent *E. coli* NEB10‐beta, and the resulting plasmid was verified by PCR for insert presence using FW pAM1‐RV *tetW* preT primers. BsaI sites between the two CRISPR direct repeats in pAM1‐preT BLC01 allowed insertion of the *tetW*‐targeting spacer using two annealed oligonucleotides with compatible overhangs (sp1 tetW top‐sp1 tetW botA). The ligation mix was transformed into *E. coli* NEB10‐beta, yielding the pAM1‐tetW CRISPR plasmid, which was verified by PCR for the spacer using FW pAM1‐RV sp1 tetW primers. For genome editing, the homology‐directed RT was cloned into the *Kpn*I‐*Nsi*I digested pAM1‐tetW CRISPR vector, forming pAM1‐tetW rep. The final construct was isolated from *E. coli* NEB10‐beta and verified by PCR for the RT using FW pAM1‐RV tetW rep primers. The cloned region between *Bgl*II and *Nsi*I sites in pAM1‐tetW rep was sequenced to confirm its integrity.

### 2.3 Transformation and Plasmid Curing

BLC01 transformation was performed according to Argnani et al. (1996) with modifications. A stationary BLC01 culture, grown anaerobically in MRS broth with 0.05% (w/v) L‐cysteine (MRS‐cys), was inoculated (1.5% v/v) into MRS‐cys supplemented with 0.2 M sucrose and incubated anaerobically at 37°C until OD_600_ ≈0.3. Cells were incubated on ice for 30 min, harvested by centrifugation (4000 *g*, 5 min, 4°C) and washed twice in electroporation buffer (0.5 M sucrose, 1 mM ammonium citrate, pH 6). The pellet was re‐suspended in 1:100 of the original volume of electroporation buffer and aliquoted into 100 μL portions. For electroporation, 1 μg of pAM1‐tetW rep plasmid was mixed with 100 μL of BLC01 competent cells. Transformation was performed using 2‐mm cuvettes under conditions of 3 kV, 25 μF and 400 Ω. Cells were recovered in 0.9 mL of MRS‐cys with 0.2 M sucrose and incubated anaerobically at 37°C for 18 h. Transformants were selected on MRS agar plates with 0.05% (w/v) L‐cysteine and 2.5 μg/mL erythromycin, incubated anaerobically at 37°C for 48–72 h. Selected transformants were inoculated in MRS‐cys with erythromycin (2.5 μg/mL) and incubated anaerobically at 37°C for 18 h. Cultures were serially diluted and plated on MRS‐cys agar and erythromycin to isolate single colonies, which were tested for tetracycline sensitivity. The *tetW* gene was PCR‐amplified from genomic DNA of tetracycline‐sensitive clones using FW tetW us‐RV tetW DS primers, and sequencing confirmed the introduced mutation.

To eliminate the pAM1‐tetW rep plasmid, *tetW*‐negative BLC01 mutants were subjected to plasmid curing. Mutants were incubated anaerobically in MRS‐cys broth at 37°C for 18 h. Cultures were then serially diluted and plated on MRS‐cys agar, followed by incubation at 37°C for 48–72 h under anaerobic conditions. Single colonies were randomly selected and replica‐plated onto MRS‐cys agar with and without 2.5 μg/mL erythromycin. Clones that failed to grow in the presence of erythromycin were identified as plasmid‐cured and stored at −80°C. One plasmid‐cured clone (BLC01‐2F3G10) was chosen for bioequivalence assessment with the parental BLC01 strain.

### 2.4 DNA Extraction, Genome Sequencing and Analysis

Biomass of BLC01 wild‐type and BLC01‐2F3G10 mutant strains, resulting from fresh cultures, was subjected to DNA extraction via the QIAcube automated extraction system (Qiagen, Hilden, Germany) using the DNeasy PowerSoil Pro Kit (Qiagen, Hilden, Germany), according to the manufacturer's instructions for Gram‐positive bacteria. The extracted DNA was quantified with the Qubit dsDNA kit (Thermo Fisher Scientific, Waltham, MA, USA), and its integrity was assessed by gel electrophoresis. Whole‐genome sequencing was performed by Synbiotec srl (Camerino, Italy) employing both the Illumina MiSeq platform, with the proprietary V3 reagents kit (read length 2 × 150 bp), and the Oxford Nanopore Technologies (ONT), using a rapid preparation kit with a R10.4.1 MinION flow cell.

The quality of the generated sequencing output was estimated, and raw reads were first analysed to exclude the presence of significant contaminations from human DNA or unrelated bacterial species using different strategies. Human contaminant reads were removed by aligning raw sequences against the GRCh38 (hg38) human genome and filtering based on alignment quality. Bacterial species contamination was assessed using MetaPhlAn 4.0 (Blanco‐Míguez et al. 2023) and Kraken v2 (Lu et al. 2022). De novo genome assembly was then performed using Flye (v0.5.0) (Kolmogorov et al. 2020), with default parameters, and the generated contigs were polished using Pilon (v1.2.0) (Walker et al. 2014). Contig number and average coverage were determined, and the quality was assessed via CheckM (v1.0.1) (Parks et al. 2015) using the most appropriate genes marker dataset as reference. Contigs with low coverage were excluded from further analysis. Functional annotation was performed using Bakta (v. 1.11.0; database version 6.0.0) (Schwengers et al. 2021). Nucleotide variation between the BLC01 and BLC01‐2F3G10 genomes was analysed using the Basic Variant Detection tool in the CLC Genomic Workbench (Qiagen, Hilden, Germany). Predicted 3D models of the wild‐type Lgt protein from *B. animalis* BLC01 and the mutant variant from BLC01‐2F3G10 were compared with NGL Viewer (Rose and Hildebrand 2015).

### 2.5 Antimicrobial Resistance Analysis

In vitro antimicrobial susceptibility analysis to eight antimicrobials was assessed by broth microdilution in accordance with ISO 10932:2010 (International Organization for Standardization 2010). Microdilution plates were incubated anaerobically at 37°C for 48 h, and minimum inhibitory concentrations (MICs) were determined spectrophotometrically at 620 nm (OD_620_). MIC values were interpreted using the microbiological cut‐off values proposed by EFSA (Rychen et al. 2018).

Growth kinetics were evaluated in the presence of lethal and sublethal tetracycline concentrations. BLC01 and BLC01‐2F3G10 were cultured at 37°C in MRS‐cys containing tetracycline at 1 μg/mL (lethal) or 0.5 μg/mL (sublethal), selected based on the previously determined antimicrobial susceptibility profiles. Cultures grown in antimicrobial‐free MRS‐cys served as controls. Bacterial growth was monitored by measuring OD_620_ at 2 h intervals over 24 h, and growth curves were generated to assess the impact of tetracycline on growth dynamics.

### 2.6 Evaluation of Probiotic Traits

Acid tolerance of BLC01 and BLC01‐2F3G10 was assessed according to Patrone et al. (2021), with minor modifications. Overnight cultures grown in MRS‐cys were harvested, washed and re‐suspended in phosphate‐buffered saline (PBS) to a standardized cell density (OD_600_ ≈0.8). Cell suspensions were exposed to PBS adjusted to pH 2, 3 and 4, with pH 7 used as a control, and incubated at 37°C. Samples collected after 1, 2 and 3 h were serially diluted, plated on MRS agar and incubated anaerobically at 37°C for 48 h to determine viable cell counts.

Bile salt tolerance was assessed following the method of Patrone et al. (2021). Standardized bacterial suspensions (OD_600_ ≈0.8) were inoculated into MRS‐cys containing 0% (control), 0.5% or 2% (w/v) ox‐bile dehydrated purified (Merck KGaA, Darmstadt, Germany) and incubated at 37°C. Viable counts were determined after 1 and 2 h of exposure by plating serial dilutions on MRS agar followed by anaerobic incubation.

Osmotic stress resistance was evaluated by assessing growth in MRS‐cys supplemented with increasing NaCl concentrations. An initial screening was performed using NaCl concentrations ranging from 1% to 5% (w/v). Based on these results, sublethal and lethal conditions (2.5%, 3% and 3.5% w/v NaCl) were selected for growth kinetic analyses. Cultures were incubated at 37°C for 24 h, and growth was monitored by measuring OD_620_ at 2 h intervals. Growth in MRS‐cys without NaCl supplementation served as the control.

EPS production was evaluated qualitatively by visual inspection of colony morphology on modified MRS agar containing glucose, fructose, sucrose or lactose as the sole carbohydrate source. Specifically, overnight cultures were plated on agar plates and incubated anaerobically at 37°C for 48 h. EPS production was assessed based on the mucoid appearance of colonies (Yadav et al. 2024).

Auto‐aggregation and co‐aggregation assays were performed as described by Rodríguez‐Sánchez et al. (2021), with slight modifications. Overnight cultures grown in MRS‐cys were harvested by centrifugation (2700 rpm for 10 min at 4°C), washed twice using sterile PBS, and re‐suspended in the same buffer to OD_620_ ≈1. Cell suspensions were incubated at 37°C without agitation, and OD_620_ of the upper phase was measured at 1, 2, 3 and 4 h to calculate auto‐aggregation percentages with the formula described by Rodríguez‐Sánchez et al. (2021).

Co‐aggregation with *E. coli* ATCC 25922 and *S. enterica* UC3605 was evaluated by mixing equal volumes of standardized bifidobacterial and pathogen suspensions. Individual bacterial suspensions served as controls. OD_620_ was monitored over 4 h of incubation at 37°C, and co‐aggregation percentages were calculated using the equation proposed by Handley et al. (1987).

### 2.7 Impact of In Vitro Gastrointestinal Digestion

In vitro static digestion was performed in accordance with the INFOGEST protocol (Brodkorb et al. 2019), which includes three sequential phases as previously described in Bozzetti et al. (2026). *Bifidobacterium* strains were cultured overnight in MRS‐cys at 37°C. The bacterial cultures were centrifuged at 13,000 rpm for 10 min, washed twice with PBS and re‐suspended in the same buffer. The concentration of the resulting cell suspension was determined by plate counting on MRS agar and was 9.31 log CFU/mL for BLC01 and 8.79 log CFU/mL for BLC01‐2F3G10. Aliquots (5 mL) of the cell suspension were used to perform the in vitro digestion. Samples were collected at the end of each digestion phase, and probiotic viability was assessed by plating serial dilutions on MRS agar (37°C, 48 h, under anaerobic conditions).

### 2.8 Adhesion to Caco‐2 and HT‐29 Cell Lines

The eukaryotic cell adhesion assays were carried out in parallel using two well‐established in vitro intestinal epithelial models: human male colon adenocarcinoma‐derived Caco‐2 cells (HTB‐37, ATCC—American Type Culture Collection, RRID: CVCL_0025) and human female colon adenocarcinoma‐derived HT‐29 cells (HTB‐38, ATCC, RRID: CVCL_0320). Certified samples of both Caco‐2 (passage 25) and HT‐29 (passage 132) cell lines were obtained through Italian IZSLER biobank (http://www.ibvr.org) in June 2023.

Cell lines were PCR‐screened on a regular basis for mycoplasma contamination by means of a commercial kit (LookOut Mycoplasma PCR Detection Kit, Merck Life Science, Milano, Italy).

These cell lines are not considered problematic concerning their possible contamination or misidentification, as they show a characteristic morphology. In any case, the cultures used for the experiment were PCR‐analysed to confirm their human origin.

Both cell lines were cultured in DMEM—Dulbecco Modified Essential Medium—supplemented with 10% (v/v) of Foetal Calf Serum (Euroclone, Pero, Italy) and 50 μg/mL of gentamicin sulphate (G1264; Sigma Aldrich, St. Louis, MO, USA) at 37°C in a humidified 5% CO_2_ atmosphere. Cells were seeded in 24‐well plates at a density of 2.5 × 10^4^ cells per well and allowed to differentiate for 2 weeks after reaching confluence, with medium changes every 48 h. Gentamicin was omitted from the culture medium 24 h prior to bacterial challenge, and cell monolayers were gently rinsed three times with PBS at 37°C immediately before infection.

The challenge was performed for BLC01, BLC01‐2F3G10 and two additional reference strains: *Lacticaseibacillus rhamnosus* ATCC 53103 (GG) as a positive control and *Lactobacillus delbrueckii* subsp. *lactis* DSM 2072 as a negative control. Adhesion assays were performed in triplicate for each strain. *Bifidobacterium* and *Lactobacillus* strains were cultivated overnight in MRS‐cys and standard MRS broth, respectively, at 37°C under anaerobic conditions. Following incubation, bacterial cells were harvested by centrifugation and washed twice with PBS buffer. Washed cells were re‐suspended and adjusted to a final concentration of 2 × 10^8^ CFU/mL. Bacterial suspensions were further diluted 1:10 in PBS, and 125 μL were added in triplicate to epithelial monolayers. Co‐incubation was performed for 1 h at 37°C in 5% CO_2_. Following incubation, monolayers were washed three times with 1 mL of PBS to remove non‐adherent bacteria.

Adhesion on Caco‐2 was assessed by microscopic visualization. Briefly, cells were fixed with 2 mL of methanol and incubated for 8 min at room temperature. Then, methanol was removed and cells were stained with 3 mL of Giemsa stain solution (1:20 in milliQ water) and incubated in the dark for 30 min at room temperature. Wells were then washed with PBS until no colour was observed in the washing solution and dried in an incubator at 30°C for 1 h. Cover glasses were then removed from the 24‐well plate, glued on the microscope slide and examined microscopically at 1000× magnification. Adhesion was expressed as the number of bacterial cells attached per 100 Caco‐2 cells based on counts from three randomly selected microscopic fields, resulting in a total of nine fields analysed per strain.

Adhesion to HT‐29 cells was quantified by real‐time PCR. After washing with PBS, cells and bacteria were detached from the well by adding 100 μL of trypsin–EDTA solution and incubating for 10 min at 37°C. Then, 400 μL of medium were added to each well and the suspensions transferred to microtubes and stored at −20°C until further processing. DNA extraction was performed on 200 μL of each sample with the bead‐technology based FastDNA SPIN kit and the Fast Prep Instrument (MP Biomedicals LLC, Irvine, CA, USA) according to the manufacturer's instructions. Then DNA was purified with ZymoBIOMICS DNA Miniprep Kit (Zymo Research Corporation, Irvine, CA, USA) following the manufacturer's recommendations. The relative quantity of bacteria with respect to eukaryotic DNA was measured by real‐time PCR. Reactions were performed on an Illumina Eco qPCR instrument with a 2× GoTaq qPCR Master Mix (Promega Corporation, Madison, WI, USA) on a final volume of 10 μL. Each sample was analysed in triplicate in a relative quantification setup, where human β‐globin gene was taken as the reference while bacterial 16S gene as the target. Primers hsGlob_Fw and hsGlob_rev were used for the amplification of the human β‐globin gene, while Uni16S_fw and Uni16S_rev to detect a region of the 16S rDNA gene conserved among most *Firmicutes* (Table 2).

### 2.9 Stability of *tetW* Mutant

To assess the genetic stability of BLC01‐2F3G10, a reversion test was performed. The mutant strain was cultured in MRS‐cys medium supplemented with tetracycline at sub‐inhibitory concentrations (0.1 and 0.5 μg/mL). As a negative control, the edited strain was grown in MRS‐cys without antimicrobial. Cultures were inoculated at 1% (v/v) and transferred every 24 h for five consecutive days. Following serial passaging, bacterial suspensions were plated on MRS agar containing increasing tetracycline concentrations (0, 1, 8, 16 and 32 μg/g) and incubated under anaerobic conditions for 48 h. The occurrence of revertant strains was evaluated based on bacterial growth on antimicrobial‐containing plates.

### 2.10 Statistical Analysis

Plate counts data were presented as mean ± standard deviation (SD). All experiments were conducted in triplicate, with the exception of acid tolerance and simulated digestion assays, which were performed in duplicate. Statistical analyses for pH, bile salt, auto‐ and co‐aggregation, simulated digestion assays were carried out using two‐way ANOVA with replicates, followed by Tukey's HSD post hoc test to assess significance (*p* < 0.05), implemented in R (version 4.4.3). Statistical analyses for adhesion to intestinal cell lines was performed using one‐way ANOVA with replicates, followed by Tukey's HSD post hoc test to assess significance (*p* < 0.05).

## 3 Results

### 3.1 Production of *tetW* Mutant

Bioinformatic analysis of *B. animalis* subsp. *lactis* BLC01 identified a Type I‐U CRISPR‐Cas system, consisting of a CRISPR array, flanked by a set of *cas* genes and including 19 spacers interspersed with a conserved 36‐nt direct repeat sequence (5′‐ATCTCCGAAGTCTCGGCTTCGGAGCTTCATTGAGGG‐3′), located immediately downstream of the *cas* operon. Spacer sequences were analysed using the CRISPRTarget webserver (Biswas et al. 2013) by searching the IMG/VR database (Paez‐Espino et al. 2017) to identify putative protospacer matches in target genomes (Table S1). The eight nucleotides upstream of each identified protospacer were extracted to determine the protospacer‐adjacent motif (PAM), revealing a conserved 5′‐CAC‐3′ sequence. The BLC01 endogenous CRISPR‐Cas system was exploited for precise genome editing by introducing a minimal self‐targeting CRISPR array along with a RT. A 33‐nt spacer (5′‐CATGCTTTTGGAGCGGCAGCGTGGGATTACCAT‐3′) was designed to target the 5′ region of *tetW* (Figure S1), immediately downstream of the 5′‐CAC‐3′ PAM sequence and was cloned into the pAM1 *E. coli–Bifidobacterium* shuttle vector (Álvarez‐Martín et al. 2008). The synthetic CRISPR array included: (i) the native leader sequence, (ii) two CRISPR direct repeats flanking the self‐targeting *tetW* spacer, and (iii) a Rho‐independent transcription terminator. Expression of this construct mimics the native CRISPR‐Cas activity, generating a crRNA that guides the endogenous Cascade‐Cas3 complex to the *tetW* locus. Genome editing was initiated by including a RT on the same plasmid, containing a mutated *tetW* sequence. The template spans 1 kb upstream and downstream of the targeted protospacer and introduces seven nucleotide substitutions that generate three consecutive premature stop codons at positions 62–64 in the TetW protein. Transformation of BLC01 with the editing plasmid resulted in transformants that initially retained tetracycline resistance. A total of 96 individual clones were screened for tetracycline sensitivity. Among them, two clones (2F3 and 2EH3) displayed a tetracycline‐sensitive (Tet^S^) phenotype. The 2F3 mutant was then cured of the editing plasmid, and whole‐genome sequencing of the BLC01‐2F3G10 cured clone was performed. Comparison with the wild‐type genome confirmed the presence of the intended mutations in *tetW*, validating both the Tet^S^ phenotype and the efficacy of reprogramming the endogenous CRISPR‐Cas system for genome editing in *Bifidobacterium* spp. (Table S2). Two additional unintended mutations were identified in the mutant strain. A deletion of a cytosine residue was identified in a non‐coding DNA region. Additionally, an amino acid substitution in the phosphatidylglycerol‐prolipoprotein diacylglyceryl transferase (Lgt) protein was determined. Specifically, an adenine‐to‐cytosine substitution led to a threonine‐to‐proline change. The mutation lies at the boundary of an α‐helix. A 3D structural analysis of the protein (Figure S2) confirmed that the overall conformation remained unaltered, suggesting that the mutation is unlikely to affect protein functionality.

### 3.2 Antimicrobial Resistance Profile and Growth Response to Tetracycline

The MICs of eight antimicrobials were determined for BLC01 and BLC01‐2F3G10 (Table 3). The MIC values of the wild‐type strain were consistent with the previously reported ranges for *B. animalis* subsp. *lactis* harbouring *tetW*, particularly for tetracycline, that exhibited a high MIC of 32 μg/mL, exceeding the EFSA microbiological cut‐off value of 8 μg/mL (Flórez et al. 2006; Gueimonde et al. 2010). Following *tetW* deletion, the tetracycline MIC decreased sharply to 1 μg/mL, falling well below the EFSA threshold, confirming the gene's contribution to the resistance phenotype. For the remaining antimicrobials, only minor variations in MIC values were observed between the two strains, and all values remained at or below the EFSA cut‐off thresholds. These results indicate that *tetW* deletion specifically affected tetracycline susceptibility without altering susceptibility to unrelated antimicrobial classes.

**TABLE 3:** Minimal inhibitory concentrations (MICs) (μg/mL) for *Bifidobacterium animalis* subsp. *lactis* BLC01 and *B. animalis* subsp. *lactis* BLC01‐2F3G10 compared to EFSA microbiological cut‐off values.

Antimicrobial	MIC BLC01 (μg/mL)	MIC BLC01‐2F3G10 (μg/mL)	EFSA cut‐off (μg/mL)
Ampicillin	2	2	2
Gentamicin	16	32	64
Kanamycin	128	128	n.r.
Streptomycin	32	32	128
Erythromycin	1	0.125	1
Clindamycin	0.25	0.063	1
Tetracycline	32	1	8
Chloramphenicol	2	1	4

Abbreviation: n.r., not required by EFSA.

To assess the physiological impact of *tetW* deletion beyond endpoint MIC determination, growth curves of both strains were monitored in MRS‐cys broth without antimicrobials and in the presence of tetracycline at sublethal (0.5 μg/mL) and lethal (1 μg/mL) concentrations for the mutant (Figure S3). In the absence of tetracycline, both strains exhibited similar growth kinetics, reaching OD_620_ values of ≈0.7–0.8 after 24 h. At the sublethal concentration, BLC01 maintained a growth profile comparable to the control, with only a slight reduction in growth rate during the exponential phase. In contrast, BLC01‐2F3G10 displayed a marked delay in exponential growth onset and a lower final OD, indicating reduced tolerance. At the lethal concentration, the mutant strain failed to grow beyond an OD_620_ of ≈0.1 throughout the 24 h period, while the wild‐type strain retained partial growth, albeit reduced compared to the control.

### 3.3 Probiotic Characterization of Wild‐Type and Mutant Strains

**FIGURE 2:**
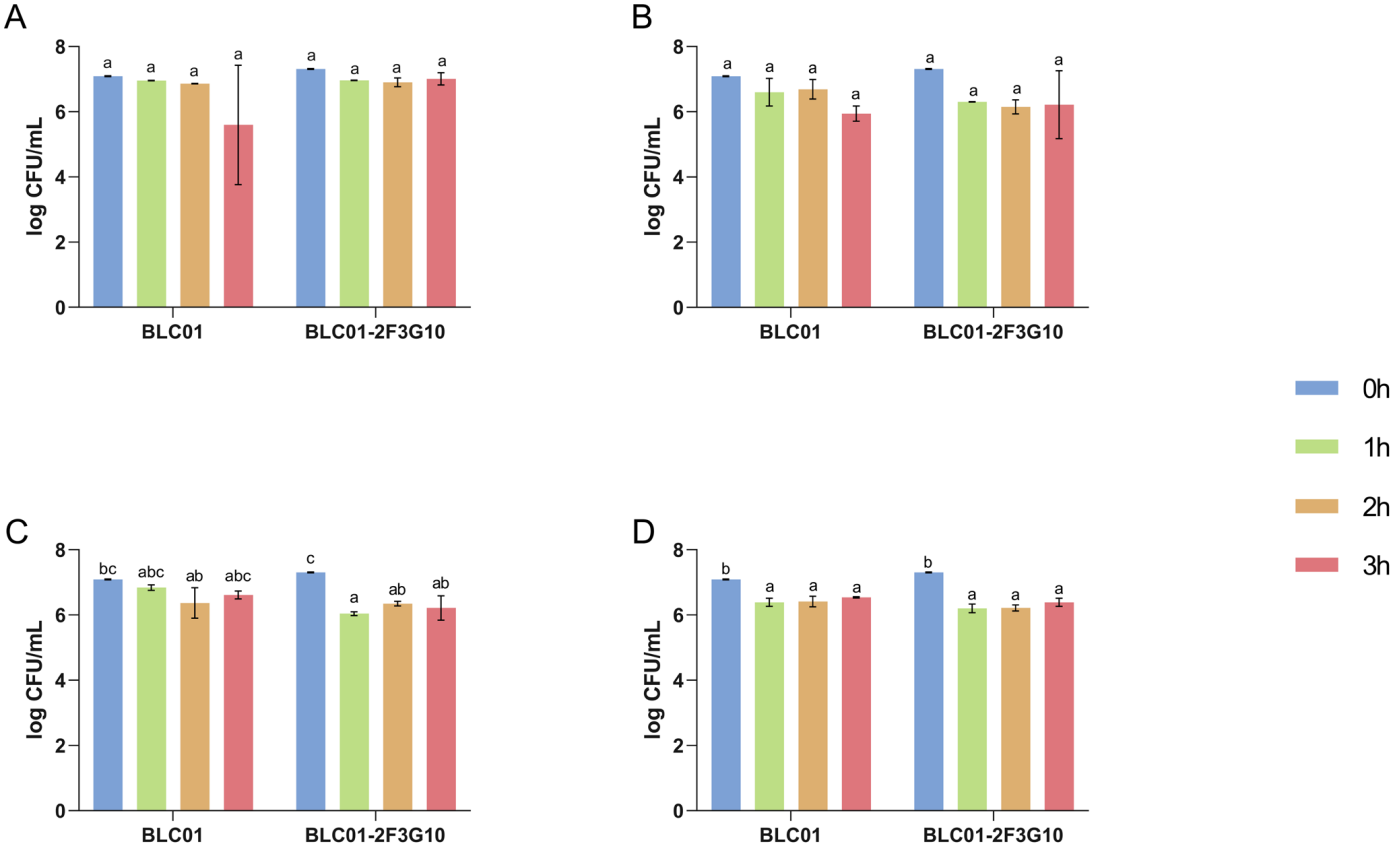
Survival of *Bifidobacterium animalis* subsp. *lactis* BLC01 and BLC01‐2F3G10 at pH 2 (A), 3 (B), 4 (C), 7 (D) as determined by viable bacteria counts. Results are presented as mean ± standard deviation (SD) (*n* = 2). Letters represent significant differences (*p* < 0.05) between samples, as determined by two‐way ANOVA followed by Tukey's post hoc test.

**FIGURE 3:**
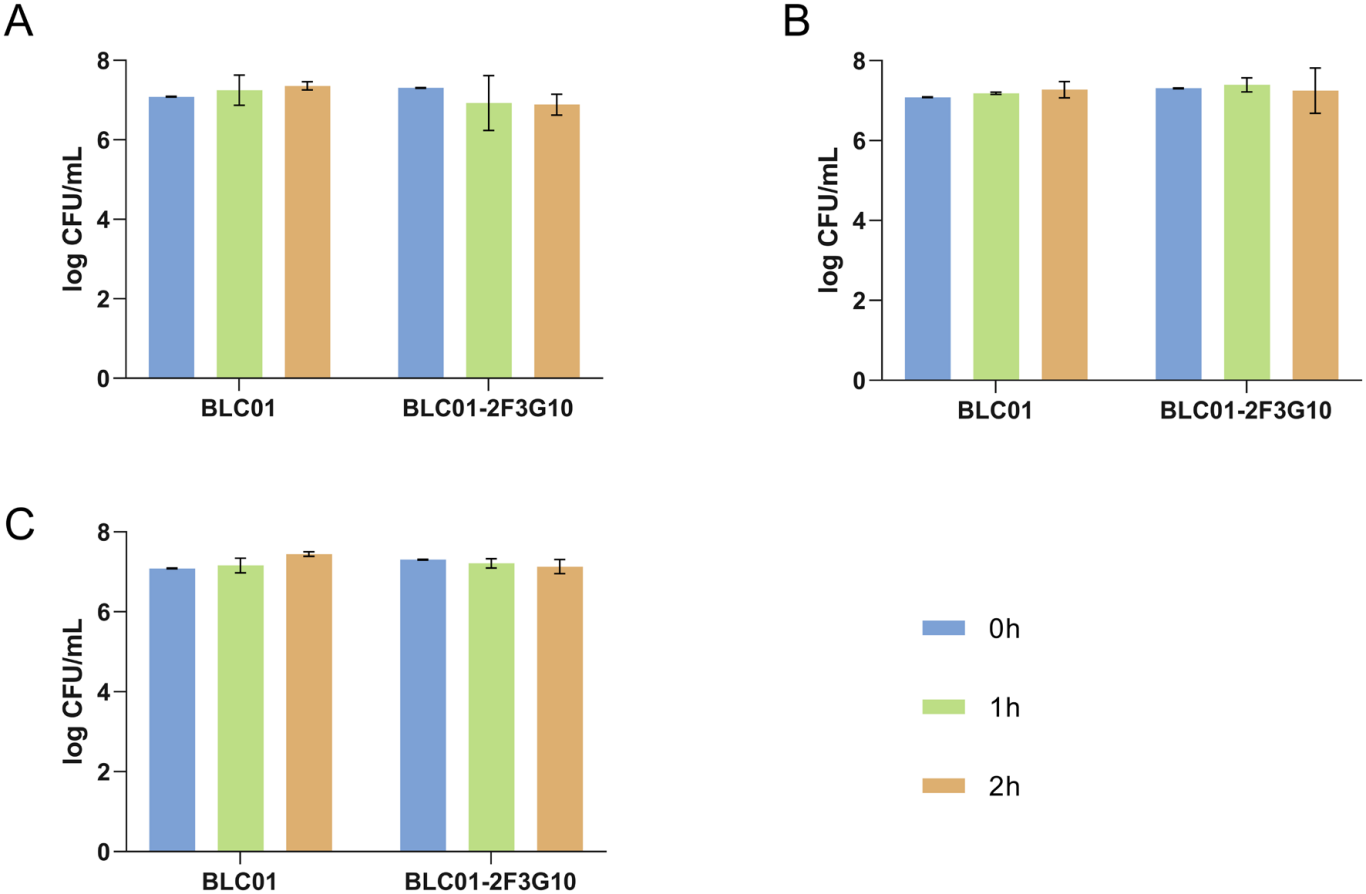
Survival of *Bifidobacterium animalis* subsp. *lactis* BLC01 and BLC01‐2F3G10 at different bile salt concentrations (A: 0%, B: 0.5%, C: 2% w/v) as determined by viable bacteria counts. Results are presented as mean ± SD (*n* = 3). No significant differences were observed among the samples, as determined by two‐way ANOVA followed by Tukey's post hoc test (*p* < 0.05).

**FIGURE 4:**
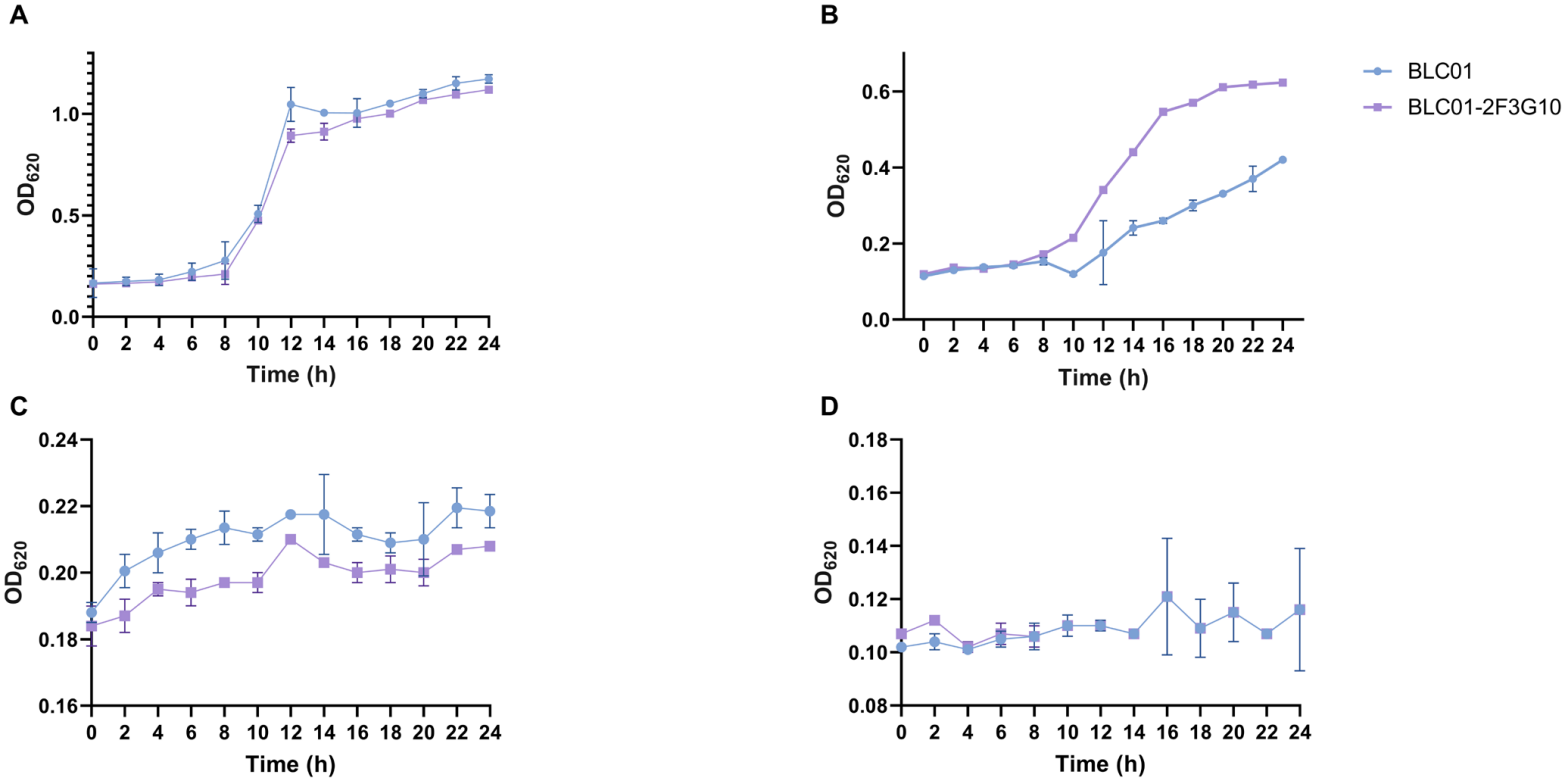
Growth curves of *Bifidobacterium animalis* subsp. *lactis* BLC01 and its mutant derivative BLC01‐2F3G10 cultivated in MRS‐cys medium supplemented with varying concentrations of NaCl: (A) 0%, (B) 2.5%, (C) 3.0% and (D) 3.5% (w/v). Growth was monitored over 24 h.

Following the generation of the *tetW*‐negative *Bifidobacterium* strain, our objective was to compare its characteristics with those of the wild‐type strain to confirm the preservation of key probiotic properties. Both BLC01 and BLC01‐2F3G10 demonstrated resistance to acidic conditions (Figure 2). After 3 h of exposure, both strains remained viable at pH 2, 3 and 4, with no statistically significant differences observed between them throughout the entire duration of the assay. Furthermore, both strains exhibited tolerance to bile salts, maintaining viability at concentrations up to 2% (w/v) (Figure 3), and no statistically significant differences were observed between the two strains throughout the entire assay period. Moreover, no pronounced differences in overall growth kinetics under osmotic stress were observed between the two strains (Figure 4). At 2.5% (w/v) NaCl, both bacteria exhibited impaired growth, as evidenced by a shortened exponential phase relative to the salt‐free control. Under this condition, the mutant strain demonstrated a modestly higher growth rate than the wild‐type, although their general growth profiles remained similar. At elevated NaCl concentrations (3.0% and 3.5% w/v), both strains experienced pronounced growth inhibition. The studied strains were also screened for their ability to produce EPS. Both *Bifidobacterium* strains were confirmed as EPS producers when grown in the presence of glucose, fructose, sucrose and lactose as sole carbon sources. No differences in colony morphology were observed between the parental strain and its mutant, indicating that the genetic modification did not affect the strain's ability to produce EPS (Figure S4).

The auto‐aggregation capacity of both strains was also evaluated, revealing strong aggregation abilities. After 1 h, BLC01 and BLC01‐2F3G10 exhibited aggregation rates of 73.11% and 73.39%, respectively. After 4 h, BLC01 reached 79.75% (a 6.65% increase), while BLC01‐2F3G10 attained 83.30%, corresponding to a 9.92% increase. No statistically significant differences were observed between the two strains in terms of their co‐aggregation behaviour. In contrast, both strains exhibited generally low and variable co‐aggregation with the gut pathogens *S. enterica* and *E. coli* .

**FIGURE 5:**
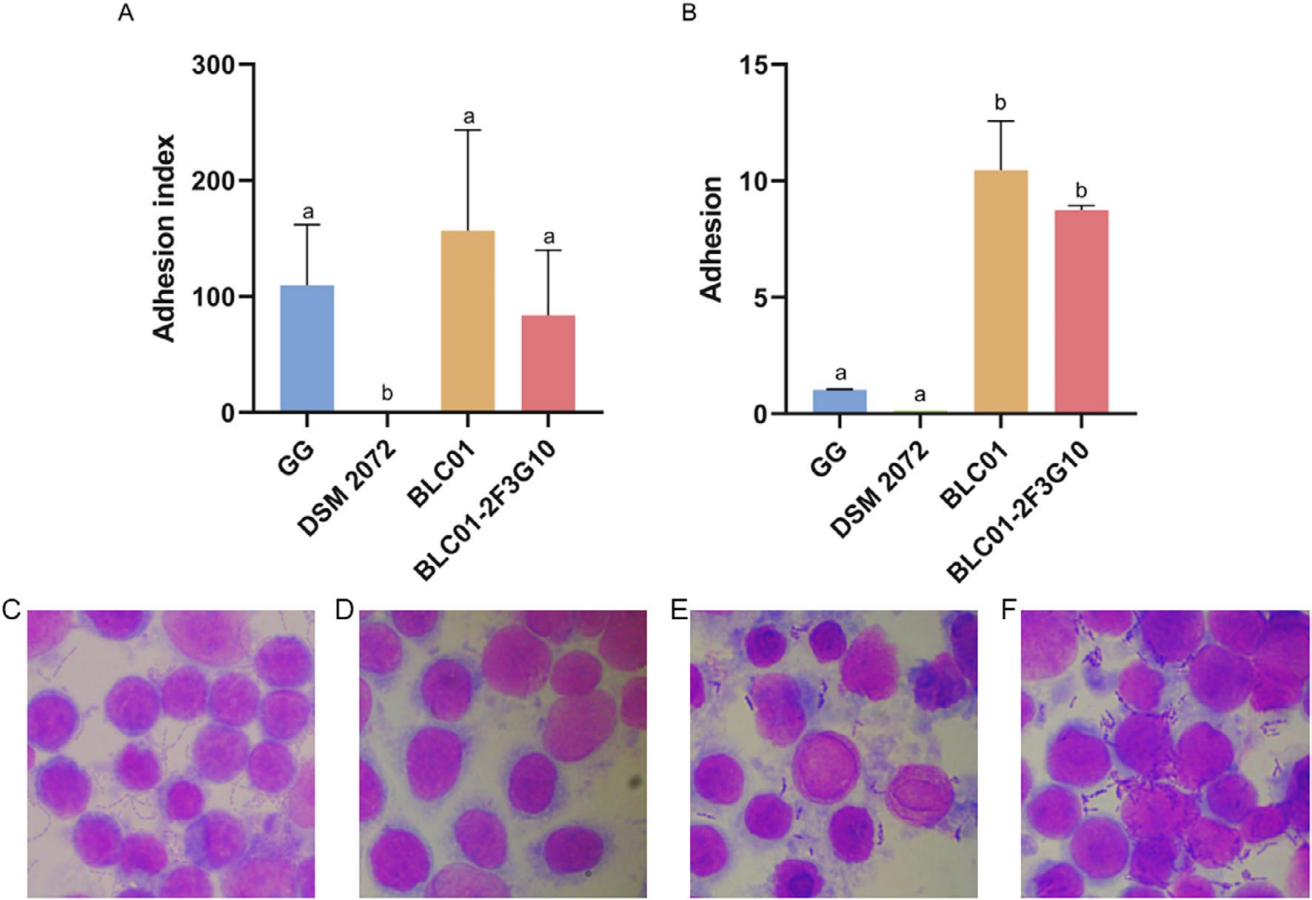
Adhesion of *Bifidobacterium animalis* subsp. *lactis* BLC01 and BLC01‐2F3G10, *L. rhamnosus* ATCC 53103 (GG), and *L. delbrueckii* subsp. *lactis* DSM 2072 to (A) Caco‐2 cell lines (evaluated by microscopic counts) and (B) HT‐29 cell lines (evaluated by real‐time PCR). Adhesion to Caco‐2 cells is expressed as the adhesion index (number of bacteria per 100 eukaryotic cells), whereas adhesion to HT‐29 cells is presented relative to *L. rhamnosus* ATCC 53103 (GG) (positive control). Data represent mean ± SD (*n* = 3). Different letters (a–b) indicate statistically significant differences between strains, as determined by one‐way ANOVA followed by Tukey's *post hoc* test (*p* < 0.05). (C–F) Representative microscopy images showing adhesion to Caco‐2 cells after staining of *L. rhamnosus* ATCC 53103 (GG) (C), *L. delbrueckii* subsp. *lactis* DSM 2072 (D), *B. animalis* subsp. *lactis* BLC01 (E), and BLC01‐2F3G10 (F).

The *Bifidobacterium* strains were also subjected to static in vitro gastrointestinal digestion following the INFOGEST protocol, and the results are presented in Figure S5. Both strains exhibited high survival during gastrointestinal transit, with no significant differences (*p* < 0.05) observed between the wild‐type strain and its mutant, or across the different digestive phases. Notably, bacterial loads consistently remained at approximately 8 log CFU/mL for both strains throughout the experiment. Additionally, both *Bifidobacterium* strains examined in this study demonstrated the ability to adhere to both Caco‐2 and HT‐29 cell lines (Figure 5). Adhesion to Caco‐2 cells did not significantly differ between the tested *Bifidobacterium* strains and the positive control, *L. rhamnosus* ATCC 53103 (GG). Notably, BLC01‐2F3G10 showed the highest adhesion level, reaching 90.56%, while the negative control, *L. delbrueckii* subsp. *lactis* DSM 2072, exhibited significantly lower adhesion (6.63%). Adhesion to HT‐29 cells was quantified using real‐time PCR, a method previously applied in Caco‐2 cell line models (Candela et al. 2005; Dingle et al. 2010). Adhesion levels were expressed relative to a randomly selected replicate of the reference strain *L. rhamnosus* ATCC 53103 (GG). BLC01 and BLC01‐2F3G10 exhibited adhesion levels that were, respectively, 10.45 and 8.74 times higher than those of GG. No statistically significant differences were observed between the two *Bifidobacterium* strains, further supporting the strains' robust performance.

### 3.4 Stability of *tetW* Mutant

To assess the stability of the edited strain, cells were cultured in the presence of tetracycline at concentrations of 0.1 and 0.5 μg/mL, which are higher than the reported minimal selective concentration (MSC) of 0.01 μg/mL (Lundström et al. 2016) and lower than the MIC. The edited *B. animalis* subsp. *lactis* strain grew normally at tetracycline concentrations of 0.1 and 0.5 μg/mL. However, no revertant colonies capable of growing at the cut‐off value of 8 μg/mL tetracycline were detected after 122 generations.

## 4 Discussions

The widespread occurrence of ARGs in probiotic strains has emerged as a major concern in the context of food safety. In particular, the widespread presence of the *tetW* gene in *B. animalis* subsp. *lactis* raises concerns regarding the potential contribution of probiotics to the transmission of ARGs at intestinal level. In this study, we reprogrammed the endogenous CRISPR‐Cas system to enable the targeted and stable inactivation of *tetW* in *B. animalis* subsp. *lactis* while preserving probiotic functionality, providing a proof‐of‐concept for a SbD strategy aligned with current regulatory expectations. The successful exploitation of a native Type I‐U CRISPR–Cas system to introduce precise stop codons into *tetW* confirms and extends previous reports on endogenous CRISPR‐based genome editing in bifidobacteria. Earlier studies primarily focused on gene deletions using endogenous systems in *B. breve* and *B. animalis* (Han et al. 2024; Pan et al. 2022), whereas the present approach relies on a minimal, well‐defined sequence modification that abolishes antimicrobial resistance without removing large genomic regions. This is particularly relevant from a regulatory and safety assessment perspective, as limited and clearly characterized edits reduce the likelihood of unintended genomic effects and facilitate molecular characterization of the edited strain (Myskja and Myhr 2020). Whole‐genome sequencing confirmed the almost complete absence of CRISPR‐associated off‐target mutations, and the single additional mutation detected in the *lgt* gene did not result in detectable alterations to the predicted structure of the Lgt protein or to the phenotypic behaviour at strain level. Although minor effects under untested conditions cannot be entirely excluded, the absence of detectable changes across multiple functional assays supports the biological neutrality of this additional mutation. This observation is consistent with previous works indicating that endogenous CRISPR systems, when properly designed, display high specificity and limited collateral effects compared with non‐targeted mutagenesis approaches (Shapiro et al. 2018). Importantly, BLC01‐2F3G10 does not retain any exogenous DNA, further strengthening its biosafety profile.

Functionally, the sharp reduction in tetracycline MIC from 32 to 1 μg/mL following *tetW* inactivation confirms the significant role of this gene in conferring resistance in *B. animalis* subsp. *lactis*, in agreement with earlier studies (Ammor et al. 2007; Gueimonde et al. 2010). Growth kinetics under sublethal (0.5 μg/mL) and lethal (1 μg/mL) tetracycline concentrations further demonstrated that the edited strain is unable to tolerate antimicrobial exposure at concentrations that do not impair the parental strain, reinforcing the effective loss of the resistance. Notably, the removal of resistance did not result in reduced fitness under antimicrobial‐free conditions, indicating that *tetW* does not contribute to basal cellular physiology. This finding aligns with previous reports indicating that ARGs in bifidobacteria are typically acquired traits, often associated with mobile genetic elements, rather than core physiological functions of the species (Wang et al. 2017).

The maintenance of stress tolerance traits is a fundamental requirement in probiotic characterization, as exposure to harsh environmental conditions during gastrointestinal transit can substantially affect viability and function (Gu et al. 2024). Survival under acidic conditions and the presence of bile salts are therefore widely regarded as a key indicator of gastrointestinal robustness (Fuochi et al. 2015). The strain BLC01‐2F3G10 exhibited acid and bile tolerance comparable to BLC01, even under conditions more stringent than those typically reported in the literature (Cizeikiene and Jagelaviciute 2021; Dosan et al. 2024; Yang et al. 2024). Given that bifidobacteria encounter fluctuating osmotic environments during both industrial processing and gastrointestinal passage (Schöpping et al. 2022), the preservation of osmotic stress tolerance further indicates that cellular mechanisms involved in osmoregulation and membrane integrity remain unaffected by the genetic modification. Consistent with these observations, both BLC01 and BLC01‐2F3G10 maintained high viability in simulated gastric and intestinal fluids. Although survival rates under such conditions are inherently strain‐dependent, previous studies on bifidobacteria consistently report substantial survivability of bifidobacteria in models mimicking the upper gastrointestinal tract, supporting the intrinsic resilience of this genus to digestive stress (Masco et al. 2007; Ziarno and Zaręba 2015). From a safety and regulatory perspective, the observed physiological equivalence between the edited and parental strains is consistent with EFSA guidance for the risk assessment of genetically modified microorganisms, where demonstrating the absence of unintended effects on key functional traits represents a central element of comparative risk evaluation (Mullins et al. 2024).

Surface‐associated properties play a pivotal role in host–microbe interactions, influencing colonization, persistence and immunomodulatory effects (Lebeer et al. 2010; Sadeghi et al. 2024). The conservation of EPS production across multiple carbohydrate sources suggests that the genetic intervention did not interfere with polysaccharide biosynthesis pathways. This observation is particularly relevant given the established contribution of EPS to stress tolerance and host interaction in bifidobacteria (Alp et al. 2010; Fanning et al. 2012; Hidalgo‐Cantabrana et al. 2014). Auto‐aggregation is considered a key factor in the adhesion of probiotic strains to the intestinal epithelium and in the competitive exclusion of pathogenic microorganisms (Lee, Jo, Seo, et al. 2024). High auto‐aggregation levels observed in both strains place them among high‐performing bifidobacteria, exceeding values commonly reported in probiotics screening studies (Cai et al. 2022; Lee, Jo, Wan, et al. 2024). Although co‐aggregation with pathogens was modest, this trait is known to be highly strain‐dependent and not necessarily predictive of in vivo efficacy, as aggregation outcomes vary substantially across strain combinations and do not consistently correlate with protective effects in physiological settings (Collado et al. 2007). Importantly, *tetW* inactivation did not introduce detectable variability or instability in aggregation behaviour, further supporting the preservation of surface‐associated functional traits.

Human in vivo studies remain the gold standard for evaluating food digestion and microbial survival; however, ethical, logistical and economic constraints often necessitate the use of simplified in vitro models (Bohn et al. 2018). When appropriately standardized, such systems provide reproducible insights into microbial behaviour under gastrointestinal stress (Treven et al. 2024). Although dynamic models better mimic physiological conditions, their technical complexity and costs limit widespread adoption (Mackie et al. 2020). In contrast, static models offer a practical and reliable approach for the preliminary assessment of probiotic robustness (Colombo et al. 2021). Among these, the INFOGEST static digestion protocol has emerged as a widely adopted reference method (Zhou et al. 2023), enabling standardized evaluation of probiotic survival under simulated gastrointestinal conditions (Rojas‐Muñoz et al. 2023; Saiz‐Gonzalo et al. 2025). BLC01 and BLC01‐2F3G10 demonstrated robust survival under simulated gastrointestinal conditions, further supporting the conclusion that *tetW* gene inactivation does not impair gastrointestinal robustness.

Adhesion to intestinal epithelial cells represents another important trait contributing to colonization and host interaction (Wang et al. 2025). In *Bifidobacterium* spp., adhesion is primarily mediated by surface‐associated proteins and other cell envelope components (Zhang et al. 2020), and enhanced adhesive capacity has been linked to immunomodulatory and anti‐inflammatory effects (Chichlowski et al. 2012). Despite the inherent limitations of in vitro adhesion assays, such models provide valuable comparative insights into adhesive potential (Rohith and Halami 2021). In this study, BLC01‐2F3G10 exhibited adhesion to Caco‐2 and HT‐29 cells comparable to that of the parental strain and the positive control, indicating that *tetW* inactivation did not impair surface‐associated traits relevant to host interaction. While the present study provides comprehensive in vitro evidence supporting the safety and functional stability of the edited strain, future in vivo studies will be required to fully validate its behaviour and persistence within the complex intestinal ecosystem.

A critical safety aspect of ARG removal strategies is the long‐term stability of the antimicrobial‐sensitive phenotype. The loss of function mediated by the introduction of stop codons is confirmed by the absence of revertants even when multiple serial MSC tetracycline concentrations were tested. By eliminating a mobile and widely conserved ARG without introducing new resistance determinants, the edited strain reduces the theoretical risk of horizontal gene transfer within the gut ecosystem. This consideration is particularly relevant given metagenomic evidence showing that probiotic‐associated tetracycline resistance genes are frequently linked to mobile genetic elements and may contribute to horizontal gene transfer within the human gut resistome (Montassier et al. 2021; Radovanovic et al. 2023; Tóth et al. 2021).

From a translational perspective, this study illustrates how NGTs can be integrated into a SbD framework for probiotic development. Rather than enhancing functionality, the applied modification addresses a recognized safety concern while preserving traits essential for probiotic efficacy. This aligns with EFSA's emphasis on comparative risk assessment and the absence of intrinsic novel hazards associated with NGT‐derived microorganisms (Mullins et al. 2024). Although demonstrated here for *B. animalis* subsp. *lactis*, this approach may be extendable to other probiotic taxa carrying well‐characterized antimicrobial resistance genes, provided that suitable endogenous CRISPR‐Cas systems are present and appropriately characterized.

In conclusion, our findings demonstrate that endogenous CRISPR‐based editing can be effectively leveraged as a precision safety tool to reshape the probiotic resistome without compromising functional performance. This strategy provides a general framework for the development of next‐generation probiotics that are both effective and aligned with evolving regulatory and societal expectations.

## Author Contributions

**Marianna Bozzetti:** conceptualization, formal analysis, investigation, methodology, writing – original draft, writing – review and editing, visualization. **Matteo Raneri:** formal analysis, investigation, methodology, writing – original draft, writing – review and editing. **Claudia Cortimiglia:** conceptualization, data curation, methodology, writing – review and editing. **Giovanni Milani:** methodology. **Federica Volontè:** funding acquisition, writing – review and editing. **Franco Lucchini:** investigation, methodology, writing – review and editing. **Daniela Bassi:** funding acquisition, conceptualization, writing – review and editing. **Pier Sandro Cocconcelli:** conceptualization, funding acquisition, supervision, writing – review and editing.

## Funding

This work was supported by National Recovery and Resilence Plan Next generation EU, OnFoods (PE00000003) and Fondazione Romeo ed Enrica Invernizzi.

## Conflicts of Interest

The authors declare no conflicts of interest.

## Supporting information


**Figure S1:** Organization of the *tetW* genetic locus in the BLC01 wt and 2F3G10 genomes. Arrows indicate complete ORFs; the direction of the arrows represents the transcription and translation direction of the ORFs. The CRISPR spacer selected to target the 5′ region of *tetW* is highlighted in yellow. The PAM sequence, recognized by the BLC01 endogenous CRISPR‐Cas system, is indicated in red. The red box depicts the *tetW* region edited in the BLC01‐2F3G10 mutant. The curly bracket encloses the nucleotide and amino acidic (aa) sequences spanning aa 1–80 of TetW. The inserted mutations (red box) result in the formation of three consecutive premature stop codons at aa 62–63–64 in the TetW sequence of the mutant.
**Figure S2:** Tertiary structure comparison of phosphatidylglycerol‐prolipoprotein diacylglyceryl transferase (Lgt) from *B. animalis* subsp. *lactis* strains. (A) Predicted 3D structure of the wild‐type Lgt protein from strain BLC01. (B) Predicted 3D structure of the mutant Lgt variant from strain BLC01‐2F3G10. (C) Structural superimposition of the wild‐type and mutant Lgt proteins reveals overall conservation of the tertiary fold. A magnified view of the mutation site shows no detectable local structural perturbations. All structural predictions, visualizations and overlays were performed using NGL Viewer.
**Figure S3:** Growth curves of *B. animalis* subsp. *lactis* BLC01 and its derivative BLC01‐2F3G10 in the absence of tetracycline (A) and in the presence of sublethal (0.5 μg/mL; B) and lethal (1 μg/mL; C) concentrations of tetracycline, as determined for the mutant strain.
**Figure S4:** Representative colony morphology of *B. animalis* subsp. *lactis* BLC01 (A) and BLC01‐2F3G10 (B) grown on modified MRS agar. Colony morphology was visually inspected after 48 h of anaerobic incubation at 37°C and used as a qualitative indicator of EPS production.
**Figure S5:** Survival of *B. animalis* subsp. *lactis* strains following static in vitro gastrointestinal digestion after oral (O), gastric (G) and intestinal (I) phases. Samples were analysed after 1 and 2 h of the gastric and intestinal phases after. Data are presented as mean ± SD (*n* = 2). No significant differences (*p* < 0.05) were observed between samples, as determined by two‐way ANOVA followed by Tukey's post hoc test.
**Table S1:** Protospacers targeted by BLC01 CRISPR‐Cas system. The 5′ protospacer flanking sequence is underlined; the PAM sequence is indicated in red.
**Table S2:** Genome comparison of BLC01 and the plasmid‐cured Tet^S^ 2F3G10 mutant, with indication of nucleotide and amino acid variations among the strains. MNV^#^, multiple nucleotide variation; SNV^#^, single nucleotide variation. Amino acid substitutions are shown using the format ‘original residue–position–mutant residue’. Positions refer to the wild‐type protein sequence.

## Data Availability

The fasta files have been deposited in the NCBI repository under the BioProject accession number PRJNA1425587 (https://dataview.ncbi.nlm.nih.gov/object/PRJNA1425587; release date: June 2026).
