# Supplementary material for: Endogenous CRISPR‐Based Removal of Tetracycline Resistance in Bifidobacterium animalis subsp. lactis Through a Safe‐by‐Design Approach
Source: Microb Biotechnol. 2026 Sep 15;19(9):e70443. doi: 10.1111/1751-7915.70443 (PMC13575638; doi:10.1111/1751-7915.70443)
Supplement: Supplementary file 1 [file MBT2-19-e70443-s001.docx]

**Table S1.** Protospacers targeted by BLC01 CRISPR-Cas system. The 5’ protospacer flanking sequence is underlined; the PAM sequence is indicated in red.

**Figure S1**. Organization of the *tetW* genetic locus in the BLC01 wt and 2F3G10 genomes. Arrows indicate complete ORFs; the direction of the arrows represents the transcription and translation direction of the ORFs. The CRISPR spacer selected to target the 5’ region of *tetW* is highlighted in yellow. The PAM sequence, recognized by the BLC01 endogenous CRISPR-Cas system, is indicated in red. The red box depicts the *tetW* region edited in the BLC01-2F3G10 mutant. The curly bracket encloses the nucleotide and amino acidic (aa) sequences spanning aa 1 to 80 of TetW. The inserted mutations (red box) result in the formation of 3 consecutive premature stop codons at aa 62-63-64 in the TetW sequence of the mutant.

**Table S2.** Genome comparison of BLC01 and the plasmid-cured Tet^S^ 2F3G10 mutant, with indication of nucleotide and amino acid variations among the strains. SNV^#^: Single Nucleotide Variation; MNV^#^: Multiple Nucleotide Variation. Amino acid substitutions are shown using the format “original residue–position–mutant residue”. Positions refer to the wild-type protein sequence.

| Position on the genome | Type of mutation | Sequence in BLC01  (3’-5’) | Sequence in BLC01-2F3G10  (3’-5’) | Gene annotation | Amino acid change (BLC01 vs BLC01-2F3G10) |
| --- | --- | --- | --- | --- | --- |
| 997734 | SNV^#^ | A | C | *lgt* (phosphatidylglycerol-prolipoprotein diacylglyceryl transferase) | Thr280Pro |
| 1179053 | MNV^#^ | GG | TT | *tetW* (tetracycline resistance protein) | Ser62Stop |
| 1179047 | MNV^#^ | CTGGA | TCACT | *tetW* (tetracycline resistance protein) | Phe63Stop_Gln64Stop |
| 1827145 | Deletion | C | - | Non-coding DNA region |  |

**Figure S2.** Tertiary structure comparison of phosphatidylglycerol-prolipoprotein diacylglyceryl transferase (Lgt) from *B. animalis* subsp. *lactis* strains. (A) Predicted 3D structure of the wild-type Lgt protein from strain BLC01. (B) Predicted 3D structure of the mutant Lgt variant from strain BLC01-2F3G10. (C) Structural superimposition of the wild-type and mutant Lgt proteins reveals overall conservation of the tertiary fold. A magnified view of the mutation site shows no detectable local structural perturbations. All structural predictions, visualizations, and overlays were performed using NGL Viewer.

**Figure S3.** Growth curves of *B. animalis* subsp. *lactis* BLC01 and its derivative BLC01-2F3G10 in the absence of tetracycline (A) and in the presence of sublethal (0.5 µg/mL; B) and lethal (1 µg/mL; C) concentrations of tetracycline, as determined for the mutant strain.

**Figure S4.** Representative colony morphology of *B. animalis* subsp. *lactis* BLC01 (A) and BLC01-2F3G10 (B) grown on modified MRS agar. Colony morphology was visually inspected after 48 h of anaerobic incubation at 37°C and used as a qualitative indicator of EPS production.

**Figure S5.** Survival of *B. animalis* subsp. *lactis* strains following static in vitro gastrointestinal digestion after oral (O), gastric (G) and intestinal (I) phases. Samples were analysed after 1 and 2 h of the gastric and intestinal phases after. Data are presented as mean ± SD (n = 2). No significant differences (p < 0.05) were observed between samples, as determined by two-way ANOVA followed by Tukey’s post hoc test.
